# Sexual Behaviour of Men and Women within Age-Disparate Partnerships in South Africa: Implications for Young Women's HIV Risk

**DOI:** 10.1371/journal.pone.0159162

**Published:** 2016-08-15

**Authors:** Brendan Maughan-Brown, Meredith Evans, Gavin George

**Affiliations:** 1 Southern Africa Labour and Development Research Unit (SALDRU), Department of Economics, University of Cape Town, Cape Town, South Africa; 2 The Institute for Humanities in Africa (HUMA), Department of Sociology, University of Cape Town, Cape Town, South Africa; 3 Health Economics and HIV and AIDS Research Division (HEARD), University of KwaZulu-Natal, Durban, South Africa; London School of Hygiene and Tropical Medicine, UNITED KINGDOM

## Abstract

**Background:**

Age-disparate partnerships are hypothesized to increase HIV-risk for young women. However, the evidence base remains mixed. Most studies have focused only on unprotected sex among women in the partnership. Consequently, little is known about other risky behaviours, such as transactional sex, alcohol use, and concurrency, as well as the behaviours of the men who partner with young women. We therefore examined differences in various sexual behaviours of both young women and their male partners by partnership age difference.

**Methods:**

We used nationally representative data from South Africa (2012) on partnerships reported by 16–24 year old black African women (n = 818) and by black African men in partnerships with 16–24 year old women (n = 985). We compared sexual behaviours in age-disparate partnerships and age-similar partnerships, using multiple logistic regression to control for potential confounders and to assess rural/urban differences.

**Results:**

Young women in age-disparate partnerships were more likely to report unprotected sex than young women in similar-aged partnerships (aOR:1.51; p = 0.014; 95%CI:1.09–2.11). Men in partnerships with young women were more likely to report unprotected sex (aOR:1.92; p<0.01; 95%CI:1.31–2.81), transactional sex (aOR:2.73; p<0.01; 95%CI:1.64–4.56), drinking alcohol before sex (aOR:1.60; p = 0.062; 95%CI:0.98–2.61), and concurrency (aOR:1.39; p = 0.097; 95%CI:0.94–2.07) when their partners were five or more years younger. The association between age-disparate partnerships and transactional sex (aOR:4.14; p<0.01; 95%CI: 2.03–8.46) and alcohol use (aOR:2.24; p<0.013; 95%CI:1.20–4.19) was only found in urban areas.

**Conclusions:**

Results provide evidence that young women’s age-disparate partnerships involve greater sexual risk, particularly through the risky behaviours of their male partners, with the risk amplified for young women in urban areas.

## Introduction

Young women in southern Africa are a critical target for HIV prevention efforts, as nearly 30% of all new infections occur among women in the 15–24 age cohort [1]. Current national strategic plans for HIV [2], and public health campaigns more generally in sub-Saharan Africa [3–5], include the prevention strategy of reducing the overall number of age-disparate partnerships (typically defined as partnerships with an age-gap between partners of 5 years or more). However, the value of this strategy has recently been called into question by a prospective longitudinal study conducted in a rural setting in KwaZulu-Natal, South Africa [2], and by the VOICE trial, involving participants from Uganda, South Africa, and Zimbabwe [6], neither of which found any significant relationship between age-disparate sex and HIV incidence for young women.

Mixed evidence on the relationship between age-disparate sex and HIV infection [2,6–11] highlights the need for a better understanding of sexual behaviours in age-disparate partnerships, and the concomitant HIV risks for young women. Previous studies have tended to focus on the sexual behaviours of women in such partnerships, rather than on the behaviours of men, although the latter play a key role in determining HIV risk for their partners. One hypothesis explaining why age-disparate partnerships may not increase HIV risk for young women has to do with the selection of older, lower-risk male partners. This might be feasible in dense, cohesive, social networks if women were to be more accurately informed about the risks associated with older compared to younger men [2]. Evidence that women have considerable agency in partner selection in sub-Saharan Africa also supports the feasibility of this hypothesis [12–15].

In addition, while previous research has established that unprotected sex is more likely in age-disparate partnerships [15–20], there is little quantitative evidence on other risk behaviours, such as transactional sex, alcohol consumption and concurrent sexual partnerships. Qualitative evidence indicates that age disparities between young women and older men amplify relationship power imbalances already present in heterosexual relationships, increasing risk of transactional sex [21–23] and violence [24–27]. Alcohol use and associated risky behaviours may also be more common in age-disparate partnerships if middle-aged men and older men drink more frequently [28]. Where concurrency is concerned, a previous South African study found that men in age-disparate partnerships are more likely to report concurrent sexual partners, thereby connecting young women in age-disparate partnerships into broader sexual networks [29].

Given that previous studies on age-disparate partnerships have typically been conducted at either a national level [16–18,29], or within urban settings [15,19,20], another important gap in our understanding is the extent to which sexual behaviour in age-disparate partnerships differs between urban and rural contexts. Research on urban-rural differences in sexual behaviour suggests that individuals living in urban areas are more likely to have current regular sex partners and have had multiple partners [30–32], but are also more likely to use condoms [31,33–36]. Significantly greater access to condoms in urban areas [36] likely explains, in part, differentials in unprotected sex across regions. Differences in access to resources and in the cohesiveness of social networks between urban compared to rural areas also make heterogeneity in sexual behaviours in age-disparate partnerships across different geographical contexts entirely plausible.

This study expands on previous research by providing a comprehensive assessment of sexual behaviour within age-disparate partnerships using nationally representative data from South Africa. We examine both the sexual behaviour of the young women and sexual behaviour of the men in age-disparate partnerships with young women. This study further extends previous work through the examination of the urban and rural differences as a component of a multidimensional assessment of sexual behaviours within age-disparate partnerships.

## Methods

### Data

Cross-sectional data were analysed from the third National HIV Communication Survey (NCS) of South Africa, conducted in 2012 and representative of the population aged 16 to 55. The NCS used a multi-stage sampling approach with stratification by province, district and area type. Primary sampling units (2001 Census and 2007 Community Survey small areas) were selected based on probability proportional to size techniques. Households, followed by individuals, were randomly selected within primary sampling units [37]. The overall response rate was 83%.

The structured face-to-face interview collected data on the respondents’ three most recent sexual partnerships and whether each partnership was on-going. We created two data sets. The first comprising all the on-going partnerships reported by women aged 16 to 24. The second comprising all the on-going partnerships reported by *male respondents* in which the reported partner was a woman 16 to 24 years old. Given that these data include all on-going partnerships (i.e. including primary and secondary partners) of participants and that the data were nationally representative, both these data sets should represent all partnerships, at the time of the survey, involving 16–24 year old women. By considering all men who reported on-going partnerships with young women, this study was able to compare the risk characteristics of older men in age-disparate partnerships to those of younger men in age-similar partnerships with the same cohort of young women. This approach enabled us to consider data that cannot accurately be reported by young women about their male partners in surveys, namely transactional sex, alcohol use and concurrency.

We restricted our data to heterosexual partnerships (98.4% and 98.7% of partnerships reported by men and women respectively) in order to explore sexual risk behaviour within age-disparate partnerships between older men and young women. We further restricted our data to populations with particularly high HIV prevalence and infection rates by focusing our analysis on black African individuals [38]. Relatively little data were excluded under this restriction as the vast majority of the original study population relevant to our analysis comprised black African women (88%) and men (86%). Without data on the race of reported partners, we made the assumption that all heterosexual partners reported were black African given the extremely high rates of same-race partnerships within South Africa [39]. Under this assumption the data reported by black African men represent partnerships involving young black African women.

### Ethics statement

The University of the Witwatersrand’s Human Research Ethics Committee and the Institutional Review Board of the Johns Hopkins Bloomberg School of Public Health granted ethical approval for the NCS, including all consent procedures. All participants provided written informed consent. The University of the Witwatersrand provided guidance on participation by children–that is, participants under the age of 18 years. For participants aged 16 and 17 years, both the child’s own consent and consent of a parent or guardian were required for participation in the study.

### Measures

Our main independent variable of interest, age-disparate partnerships, identify partnerships with a 5-year or greater age disparity between young women and older men by calculating the difference between the participants’ age and their partner’s age. Four binary outcome variables measure sexual behaviour that could increase HIV infection risk for young women. Unprotected last sex identifies individuals who reported not using a condom the last time they had sex with their partner. The transactional sex variable measures whether women reported *receiving* gifts or money from their partner in the past year in exchange for sex and whether men reported *giving* their partner gifts or money in exchange for sex. Alcohol at last sex indicates respondents who reported drinking alcohol before they last had sex with their partner. The concurrency variable identifies men who reported two or more on-going partnerships (i.e. they expected to have sex again with the partners.) (See S1 Text for the wording of the survey questions.)

Individual-level factors that could potentially influence both the outcome measure and age-disparate partnerships included in the analysis as control variables were: age; being born outside of South Africa; education (categorised only as <grade 12 or ≥ grade 12 due to the lack of variation in other categories recorded); employment status; and household wealth (derived from summing “yes”/ “no” responses to household ownership of seven designated assets). We also accounted for two HIV-related factors: having ever tested for HIV and knowledge about HIV according to a sum of correct responses to five questions (see S1 Text). Partnership specific control variables included partnership type (married, cohabiting, casual, or other); partnership duration (less than a month, 2–6 months, 6–12 months, 1 year and more); and whether the respondents reported knowing their partner’s HIV status.

### Analysis

First, individual-level data were used to describe the men and women in the samples. All subsequent analyses were conducted using the partnership as the unit of observation. We used standard differences in proportions tests to compare the prevalence of sexual behaviours in age-disparate and age-similar partnerships, with the analysis conducted separately by rural and urban setting. Our analysis of concurrency was restricted to partnerships reported by men because we are interested in how behaviour *within* a partnership impacts HIV risk for young women. At the partnership level, concurrency is not an additional risk factor for the person who has concurrent partners, because the risk comes simply from having multiple partners [40]. Concurrency is, however, a risk for the partner of the person who has concurrent relationships because those who have concurrent sexual partners may acquire HIV outside of the partnership and transmit HIV to their partner [40]. Therefore, young women are at a greater risk for HIV infection if the men they partner with have concurrent partners. The concurrency analysis accordingly assesses the proportion of men in partnerships with young women who reported an overlapping partner (of any age) at the time of the interview.

Multiple logistic regression models were created to assess the association between sexual behaviours and partnership type (age-similar v age-disparate), controlling for potential confounders. To investigate differences between rural and urban settings we reran our regression analysis and included an interaction term (created by multiplying an indicator of living in a rural setting by the age-disparate variable).

All analyses were adjusted to account for the complex study design (i.e. we used weighted data for all analyses and where relevant adjusted for stratified and clustered sampling) and non-response. By accounting for clustering using primary sampling units the analysis controls for all potential within-cluster error correlation, including potential correlation between partnership data [41]. This is important as some participants reported more than one partnership and an individual’s sexual behaviour across partnerships might be correlated. A priori we set p<0.1 to denote statistical significance given that the coefficients in the regression analysis would be estimated off relatively small numbers when disaggregating data by both partnership type and geographic location [42].

We conducted two sensitivity analyses. First, we assessed whether a different measure of unprotected sex (inconsistent condom use within partnerships) altered findings. Second, we tested whether using a continuous independent variable (years age difference between partners) resulted in different findings.

## Results

Table 1 displays descriptive statistics for the sample of 16 to 24 year old women (n = 790) and the sample of men who reported being in a partnership with 16 to 24 year old women (n = 801). The majority of the sample of women (77%) was between the ages of 20 and 24. Among the male sample, 53% were within the same age-bracket as the female sample (16–24), while a relatively low proportion of men were 30 years or older (13%). Just over half of each sample was from an urban setting. Young women reported 818 on-going partnerships, with 41% involving an age-disparate partner (5 years or older). Men reported 985 on-going partnerships involving 16 to 24 year old women, with 37% age-disparate. Differences in the proportion of age-disparate partnerships in urban and rural areas were small.

**Table 1:** Sample characteristics.

	Women (16–24 years old)	Men in partnerships with 16–24 year old women
**Individual level data**	n = 790	n = 801
Age (mean)	21	24.6
Age categories		
16–19	23%	11%
20–24	77%	42%
25–29		34%
30+	na	13%
Grade 12 complete	52%	55%
Not enough to eat (often/sometimes/rarely in past 12 months)	32%	33%
Employed	13%	13%
Urban	55%	52%
Know someone who died of AIDS	54%	55%
Had an HIV test	85%	57%
Good HIV knowledge (4 or 5 of 5 correct answers)	81%	80%
**Partnership data**	n = 818	n = 985
Age disparate (5+ age-gap)	41%	37%
Partnership age difference 10+ years	7%	11%
Age disparate in urban areas	41%	40%
Age disparate in rural areas	40%	35%
Married	13%	10%

Notes: All figures are adjusted to account for the complex study design and non-response.

### Sexual behaviours reported by women

**Fig 1:**
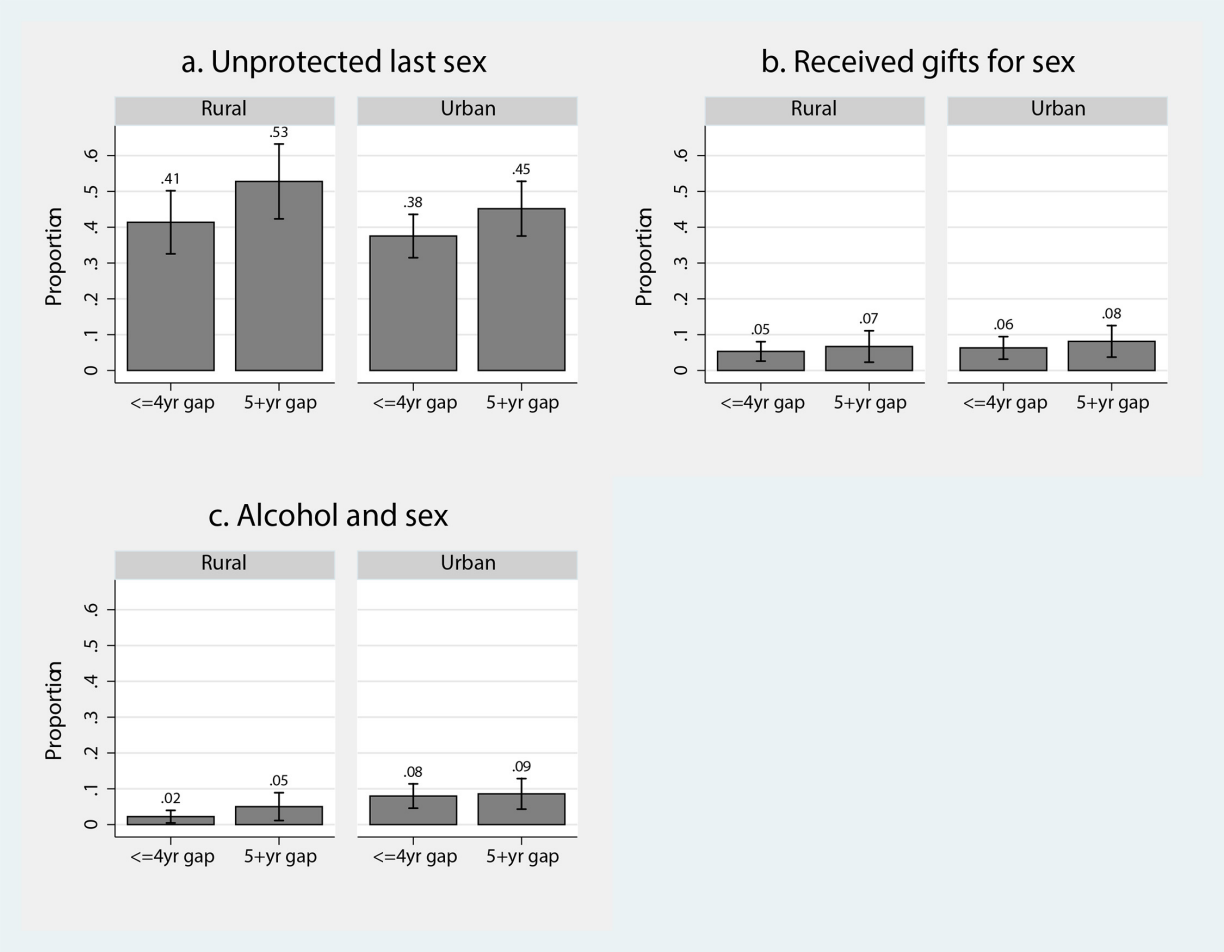
Sexual behaviours reported by 16–24 year old women by partnership type (age-disparate v age-similar) and geographic (urban/rural) setting. 1a: unprotected last sex; 1b: received gifts for sex; 1c: alcohol and sex.

Fig 1 displays sexual behaviours in age-disparate and age-similar partnerships as reported by women in both rural and urban settings. Condom use was less common in age-disparate partnerships. Unprotected sex was 11 percentage points higher in age-disparate partnerships in rural areas (p = 0.068) and 8 percentage points higher (p = 0.130) in urban areas. No obvious differences between partnership type and transactional sex or alcohol use were evident in either urban or rural settings.

Multiple regression analysis of sexual behaviours reported by women (Table 2) found that, after accounting for confounders, the patterns between partnership type and location of residence remained consistent with those in Fig 1. Panel A presents results from the models without the interaction term and Panel B the results from models including the age-disparate times rural interaction term. Table 2, Panel A shows that the odds of unprotected sex (A1) were significantly higher in age-disparate partnerships (aOR: 1.51; p = 0.014; 95%CI: 1.09–2.11), but the associations between age-disparate partnerships and transactional sex (A2) and alcohol use (A3) were not statistically significant. See S1 Table for the full model.

**Table 2:** Multiple logistic regression models of sexual behaviours within partnerships reported by 16 to 24 year old women.

Dependent variable:	Unprotected last sex	Received gifts for sex	Alcohol and sex
**Panel A**	A1	A2	A3
	aOR (95% CI)	aOR (95% CI)	aOR (95% CI)
Age-disparate	1.51**	1.20	1.30
	(1.09–2.11)	(0.65–2.21)	(0.74–2.29)
Rural	0.92	0.72	0.54
	(0.61–1.38)	(0.30–1.73)	(0.19–1.56)
Control variables	Yes	Yes	Yes
n	816	785	780
**Panel B**	B1	B2	B3
	aOR (95% CI)	aOR (95% CI)	aOR (95% CI)
Age-disparate	1.44	1.20	1.02
	(0.93–2.24)	(0.53–2.73)	(0.54–1.95)
Age-disparate*Rural	1.12	0.99	2.40
	(0.55–2.25)	(0.26–3.80)	(0.57–10.17)
Rural	0.88	0.72	0.35
	(0.55–1.40)	(0.25–2.11)	(0.07–1.62)
Control variables	Yes	Yes	Yes
n	816	785	780

Notes

*** p<0.01

** p<0.05

* p<0.1.

95% Confidence Intervals in brackets. Control variables include age, education, employment status, household wealth, HIV testing history, HIV knowledge, partnership type, partnership length and knowledge of partner’s HIV status (see S1 Table and S2 Table for the full models including coefficients for all control variables). All analyses are adjusted to account for the complex study design and non-response.

In Panel B, the coefficient on the interaction term (age-disparate*rural) was statistically insignificant in all models. These data indicate that the association between sexual behaviour reported by women and age-disparate partnerships did not differ significantly between rural and urban areas. (See S2 Table for the full model, and S3 Table, which shows similar results when using ordinary least squares regression models).

### Sexual behaviours reported by men

**Fig 2:**
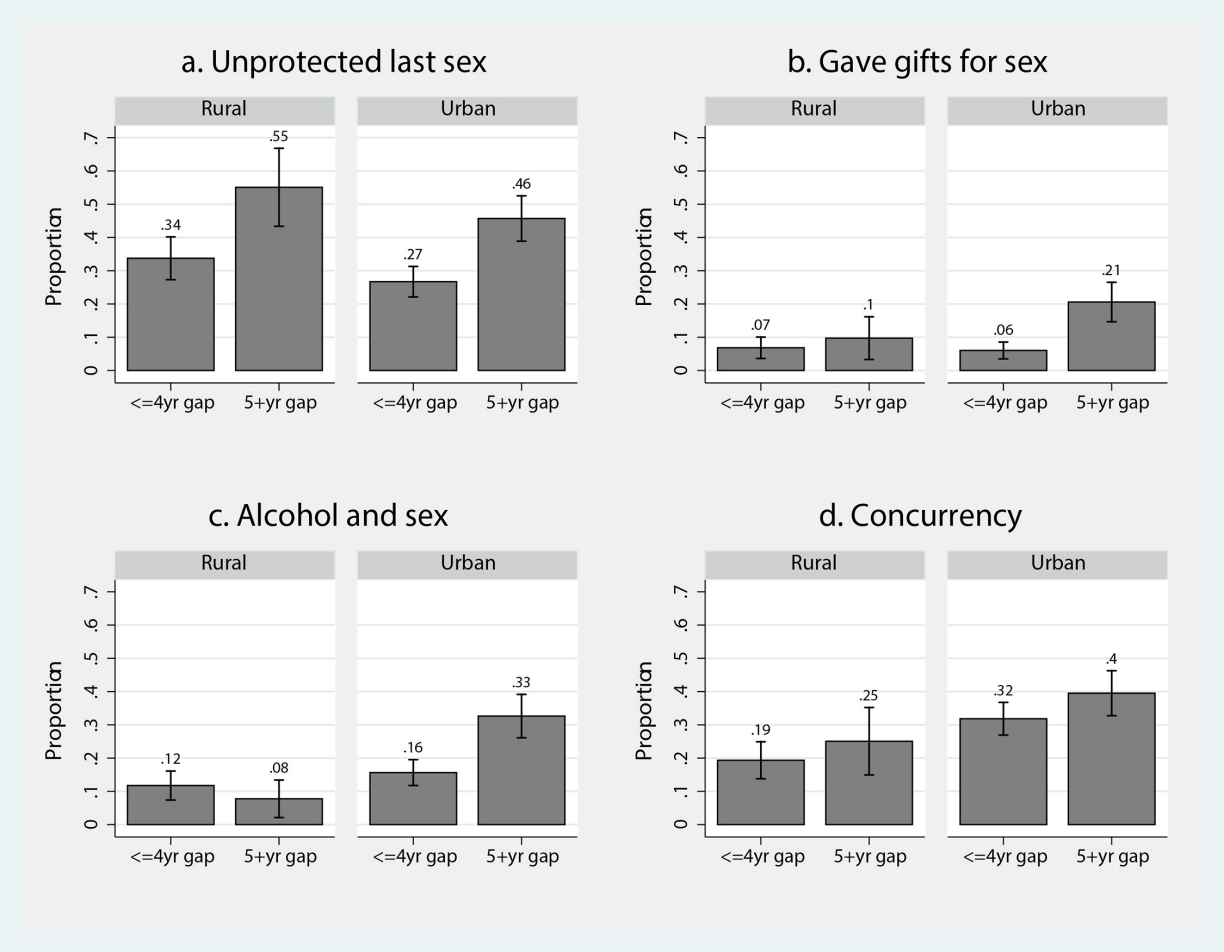
Sexual behaviours reported by men in partnerships with 16–24 year old women by partnership type (age-disparate v age-similar) and geographic (urban/rural) setting. 2a: unprotected last sex; 2b: gave gifts for sex; 2c: alcohol and sex; 2d: concurrency.

Fig 2 presents the sexual behaviour data reported by men in partnerships with 16 to 24 year old women. Fig 2A shows, consistent with data reported by women in Fig 1, that age-disparate partnerships were more likely to involve unprotected sex than similar-aged partnerships. Differences in condom use by partnership type were similar in rural and urban areas: unprotected last sex was 21 percentage points higher in age-disparate partnerships in rural areas (p = 0.012) and 19 percentage points higher in age-disparate partnerships in urban areas (p<0.01). Transactional sex (Fig 2B) was only slightly higher in rural age-disparate partnerships (+2.9% points, p = 0.375), but significantly greater in age-disparate partnerships in urban settings (+15% points, p<0.01). The association between age-disparate partnerships and drinking alcohol before last sex (Fig 2C) varied between settings. In rural areas, a negative, but not statistically significant, relationship was found between age-disparate partnerships and drinking alcohol before last sex (-4% points, p = 0.170), while in urban areas men in age-disparate partnerships were significantly more likely to report this behaviour (+17% points, p<0.01). Fig 2D shows that a greater proportion of men in age-disparate partnerships with young women reported concurrency than men in similar-aged partnerships, but the differences were not statistically significant (rural: +6% points, p = 0.30; urban: +8% points, p = 0.14).

Table 3 presents the results from multiple regression models of sexual behaviours of men within partnerships involving young women. Panel A presents results from the models without the interaction term and Panel B the results from models including the age-disparate times rural interaction term. The models in panel A show that age-disparate partnerships were associated with higher levels of unprotected last sex (A1: aOR:1.92; p<0.01; 95%CI: 1.31–2.81), transactional sex (A2: aOR:2.73; p<0.01; 95%CI: 1.64–4.56), alcohol use before sex (A3: aOR:1.6; p = 0.062; 95%CI: 0.98–2.61) and concurrency (A4: aOR: 1.39; p = 0.097; 95%CI: 0.94–2.07). Additional analysis found that the positive association between age-disparate partnerships and concurrency was particular large in partnerships involving younger (16 to 19 year old) women (aOR: 2.62; p<0.01; 95%CI: 1.29–5.3).

**Table 3:** Multiple logistic regression models of sexual behaviours reported by men in partnerships with 16 to 24 year old women.

Dependent variable:	Unprotected last sex	Gave gifts for sex	Alcohol and sex	Concurrency
**Panel A**	A1	A2	A3	A4
	aOR (95% CI)	aOR (95% CI)	aOR (95% CI)	aOR (95% CI)
Age-disparate	1.92***	2.73***	1.60*	1.39*
	(1.31–2.81)	(1.64–4.56)	(0.98–2.61)	(0.94–2.07)
Rural	1.03	0.90	0.51**	0.52**
	(0.73–1.48)	(0.47–1.73)	(0.29–0.89)	(0.31–0.86)
Controls	Yes	Yes	Yes	Yes
n	980	961	966	982
**Panel B**	B1	B2	B3	B4
	aOR (95% CI)	aOR (95% CI)	aOR (95% CI)	aOR (95% CI)
Age-disparate	1.70***	4.28***	2.38***	1.28
	(1.15–2.52)	(2.25–8.13)	(1.28–4.46)	(0.80–2.07)
Age-disparate*Rural	1.29	0.34**	0.27**	1.22
	(0.55–3.05)	(0.12–0.92)	(0.10–0.74)	(0.54–2.79)
Rural	0.94	1.55	0.84	0.48**
	(0.60–1.48)	(0.67–3.60)	(0.46–1.53)	(0.25–0.90)
Controls	Yes	Yes	Yes	Yes
n	980	961	966	982

Notes

*** p<0.01

** p<0.05

* p<0.1.

95% Confidence Intervals in brackets. Control variables include age, education, employment status, household wealth, HIV testing history, HIV knowledge, partnership type, partnership length and knowledge of partner’s HIV status (see S4 Table and S5 Table for the full models including coefficients for all control variables). All analyses are adjusted to account for the complex study design and non-response.

The coefficients on the interaction terms in models B1 and B4 (Table 3, Panel B) are close to one and not statistically significant, indicating no differences between rural and urban areas in the association between age-disparate partnerships and either condom use or concurrent sexual partnerships, respectively. In contrast, large urban-rural differences were found regarding the association between age-disparate partnerships and transactional sex and alcohol use. The coefficient on the interaction term indicated that the association between age-disparate partnerships and both transactional sex (B2: aOR: 0.34; p<0.05; 95%CI: 0.12–0.92) and drinking before sex (B3: aOR: 0.27; p<0.05; 95%CI: 0.10–0.74) was significantly weaker in rural areas. Results from this analysis were substantively similar when ordinary least square regression models were used instead of logistic regression models: see S6 Table). Consistent with these findings, additional analysis (see S7 Table) found that the association between age-disparate partnerships and transactional sex was highly significant *within* urban areas (aOR: 4.14; p<0.01; 95%CI: 2.03–8.46), but not significantly different *within* rural areas (aOR: 1.04; p = 0.92; 95%CI: 0.42–2.58). Similarly, men in age-disparate partnerships within urban areas were more likely to have consumed alcohol before sex than men in similar-aged partnerships (aOR: 2.24; p<0.012; 95%CI: 1.20–4.19), while this was not the case in rural areas (aOR: 0.8; p = 0.579; 95%CI: 0.35–1.81).

Sensitivity analyses (see S8 Table and S9 Table) found that age-disparate partnerships were also associated with inconsistent condom use during the partnership as reported by women (aOR: 1.42; p = 0.034; 95%CI: 1.03–1.98) and by men in partnerships with 16–24 year old women (aOR: 1.81; p<0.01; 95%CI: 1.32–2.47). In addition, all statistically significant and positive associations between our binary measure of age-disparate partnerships and risky sexual behaviour remained statistically significant and positive in models using a continuous independent variable (years age difference between partners; see S10 Table and S11 Table).

## Discussion

The relationship between age-disparate partnerships and HIV infection risk among young women is complex and not fully understood. This study expands on previous research by assessing a broad range of sexual behaviours within age disparate partnerships as reported by both young black African women and the black African men in partnerships with young women.

Consistent with previous studies from South Africa [10], age-disparate partnerships were common among young women, with a little over a third of young women’s on-going partnerships involving a male partner five or more years older. This result was consistent in the partnership data reported by 16 to 24 year old women and the data reported by male respondents in partnerships with 16 to 24 year old women, which instils confidence that both samples are representative of age-disparate partnerships involving young women.

Overall, our findings show that age-disparate partnerships are associated with behavioural characteristics which could increase the risk of HIV infection for young women. Consistent with previous studies [15–20], condoms were found less likely to be used in age-disparate compared to age-similar partnerships. This finding was substantively similar in partnership data collected among young women and among men in partnerships with young women. Since gender biases have been observed in self-reporting of sexual behaviour [43], our findings provide strong evidence that age-disparate partnerships involve more unprotected sex.

In line with qualitative research that indicates transactional sex is a motivating factor for age-disparate partnerships involving young women [14,44,45], we found a strong, positive association between age-disparate partnerships and transactional sex, as reported by men in partnerships with young women. While a greater proportion of age-disparate partnerships in our data reported by men were classified as spousal relationships (21% of age-disparate, 7% of age-similar), as has been found elsewhere in South Africa [46], only a small proportion of all partnerships involved married partners and the association between age-disparate partnerships and transactional sex remained robust after controlling for marital status. However, despite evidence that transactional sex–motivated both by subsistence needs and conspicuous consumption–plays a role in age-disparate relationship formation in urban [23,24,47–49], as well as in rural settings in southern Africa [22,44,50], our results indicate that age-disparate partnerships are more likely to involve transactional sex only in urban areas.

The explanation for this geographic variation is unclear. Such variation may reflect differences between rural and urban areas in gendered material inequity, which has been established as a key driver of transactional sex [25,51]. Such variation could also be due to urbanisation which has both reshaped masculinities and sexual practices [51] and created differentials between rural and urban areas in the degree to which young women are subjected to traditions that guide relations between generations and between men and women [25]. Another potential explanation is that this variation is a confluence of the more prevalent practice of multiple sexual partnering found in urban than rural areas [30–32], which is also reflected in our results, and the positive association between transactional sex and multiple sexual partners [52,53].

Regardless of the mechanism underlying the geographic variation, our findings suggest greater sexual risk for young women in age-disparate partnerships in urban areas as transactional sex has been linked to high risk behaviours including unprotected sex [14] and anal sex [54,55], as well as intimate-partner violence [56–58]. Gender differences in perceptions about motivations and intentions within partnerships in South Africa [56,59,60] align with our findings that men are more likely to perceive a transactional element in partnerships than women. HIV infection risk may be especially high in age-disparate partnerships in cases where men perceive there to be a transactional element to the partnership while women do not. For instance, young women are less likely to use condoms with men from whom they expect monogamy and commitment [61,62]. Further research is required to explore how gendered perspectives of transactional partnerships influence HIV risk.

Alcohol use is also associated with high-risk sexual behaviours [63]. We found that men in partnerships with young women in urban settings were significantly more likely to drink alcohol before sex when the partnerships were age-disparate. Gender differences in risk associated with alcohol use have been reported elsewhere in southern Africa, with a woman’s risk often associated with her male partner’s consumption of alcohol [64]. Our results suggest that this risk is greater for women in urban areas in age-disparate partnerships.

Building on a nascent literature [29], our results show that concurrency among men may be an additional factor associated with age-disparate partnerships that increases young women’s HIV risk. Our findings indicate that young women with age-disparate male partners are more likely to be connected to a broader sexual network, and therefore more likely to acquire HIV, than women in age-similar partnerships. While the strength of this association was relatively weak (only borderline significant at the 10% level) among all partnerships involving 16–24 year old women–indicating the need for further research to tests this relationship–concurrency was identified as a particularly strong risk factor associated with age-disparate partnerships among adolescent (16 to 19 year old) women. Currently, theories on how age-disparate partnerships increase HIV risk for young women are closely tied to the statistical likelihood that older partners are more likely to be HIV positive than younger partners [38,65]. As a result of concurrency among men, age-disparate partnerships could also increase a young women’s HIV risk even when her partner is HIV-negative at the start of the partnership.

Results from this study have several policy and research implications. Our findings provide the first evidence indicating that the HIV infection risk for young women associated with age-disparate partnerships may be greater in urban than in rural settings. Explanations for differences in sexual behaviour in age-disparate partnerships between urban and rural areas should be explored. Our evidence supports interventions to reduce the HIV infection risk that age-disparate partnerships pose to young women, and points towards such interventions being particularly important in urban areas. Our finding that age-disparate partnerships are associated with greater sexual risk, even in rural areas, does not support the theory that careful selection of older, lower-risk male partners may mitigate the HIV risk associated with age-disparate partnerships. Alternative theories for the lack of association between age-disparate partnerships and HIV risk found in some studies [2,6] should be explored. For example, differential uptake of antiretroviral therapy by age, with older men living with HIV more likely to be on ART [38], and less likely to be lost to follow-up after HIV diagnosis compared to younger men [66], may mitigate the HIV infection risk that age-disparate partnerships pose for young women.

Limitations in our study include the potential for misreporting of partnerships [67], and the potential for error in reporting of partner’s age [68]. Social desirability bias may have influenced self-reported measures of sexual behaviour. Our measure of transactional sex may not fully capture transactional elements within partnerships, which can be more nuanced than the direct exchange of money and gifts for sex. For example, a study found that one in three U.S. women have had economically motivated relationships, yet only one in ten of those women reported exchanging sex for money [69]. Furthermore, compared to our results, higher levels of transactional sex were found in rural South Africa in a study using a multidimensional measurement of transactional sex [70]. In addition, our data is from a household survey and may not accurately represent migrant workers and mobile populations. Finally, our results may be generalized to partnerships of young black African women, but not to other population groups within South Africa.

In conclusion, we found that young women’s age-disparate partnerships in both urban and rural settings are characterised by greater sexual risk behaviour. This is especially the case in urban areas. Results from this study support the hypothesis that age-disparate partnerships increase HIV infection risk for young women and suggest that interventions to reduce this risk are warranted. Future research and policy development should be cognisant that age-disparate partnerships in urban areas may involve additional elements of risk for young women.

## Supporting Information

S1 Table Full multivariable logistic regression results for the models presented in Table 2, Panel A. (DOCX)

S2 Table Full multivariable logistic regression results for the models presented in Table 2, Panel B. (DOCX)

S3 Table Ordinary Least Squares regression models of sexual behaviours in partnerships reported by 16 to 24 year old women, with the inclusion of the interaction term ‘age-disparate*rural’. (DOCX)

S4 Table Full multivariable logistic regression results for the models presented in Table 3, Panel A. (DOCX)

S5 Table Full multivariable logistic regression results for the models presented in Table 3, Panel B. (DOCX)

S6 Table Ordinary Least Squares regression models of sexual behaviours reported by men in partnerships with 16 to 24 year old women, with the inclusion of the interaction term ‘age-disparate*rural’. (DOCX)

S7 Table Multivariable logistic regression models of transactional sex and alcohol consumption *within* rural and urban areas as reported by men in partnership with 16–24 year old women. (DOCX)

S8 Table Multivariable logistic regression models of inconsistent condom use within partnerships reported by 16 to 24 year old women. (DOCX)

S9 Table Multivariable logistic regression models of inconsistent condom use reported by men in partnerships with 16 to 24 year old women. (DOCX)

S10 Table Multivariable logistic regression models of sexual behaviours within partnerships reported by 16 to 24 year old women (independent variable = years age difference). (DOCX)

S11 Table Multivariable logistic regression models of sexual behaviours reported by men in partnerships with 16 to 24 year old women (independent variable = years age difference). (DOCX)

S1 Text Relevant questions for our analysis from the Third National Communication Survey, 2012. (DOCX)
