# Supplementary material for: Sexual Behaviour of Men and Women within Age-Disparate Partnerships in South Africa: Implications for Young Women's HIV Risk
Source: PLoS One. 2016 Aug 15;11(8):e0159162. doi: 10.1371/journal.pone.0159162 (PMC4985138; doi:10.1371/journal.pone.0159162)
Supplement: S1 Table — (DOCX) [file pone.0159162.s001.docx]

**S1 Table.** Full multivariable logistic regression results for the models presented in Table 2, Panel A.

|  | A1 | A2 | A3 |
| --- | --- | --- | --- |
| VARIABLES | Unprotected last sex | Received gifts for sex | Alcohol and sex |
|  |  |  |  |
| Age disparate (vs similar-aged) | 1.51** | 1.20 | 1.30 |
|  | (1.09 - 2.11) | (0.65 - 2.21) | (0.74 - 2.29) |
| Rural | 0.92 | 0.72 | 0.54 |
|  | (0.61 - 1.38) | (0.30 - 1.73) | (0.19 - 1.56) |
| Age (16-24) | 1.07 | 1.09 | 1.06 |
|  | (0.98 - 1.16) | (0.92 - 1.28) | (0.89 - 1.25) |
| Born in South Africa | 0.59 | 0.54 |  |
|  | (0.18 - 1.99) | (0.13 - 2.31) |  |
| Completed Grade 12 | 0.70* | 1.44 | 0.97 |
|  | (0.48 - 1.02) | (0.68 - 3.08) | (0.47 - 1.97) |
| Employed (base = no) |  |  |  |
| Employed | 1.03 | 0.73 | 0.51 |
|  | (0.63 - 1.70) | (0.27 - 2.00) | (0.18 - 1.46) |
| Missing data | 1.61 | 2.54 | 0.13* |
|  | (0.41 - 6.22) | (0.22 - 29.47) | (0.01 - 1.39) |
| Assets (0-7) | 0.90** | 0.89 | 1.21** |
|  | (0.83 - 0.99) | (0.75 - 1.05) | (1.02 - 1.42) |
| HIV tested (base = “no”) |  |  |  |
| Been tested | 1.22 | 0.30*** | 0.59 |
|  | (0.75 - 1.97) | (0.14 - 0.64) | (0.26 - 1.34) |
| Missing data | 2.20 | 4.35 | 0.75 |
|  | (0.40 - 12.01) | (0.68 - 27.81) | (0.06 - 9.70) |
| HIV knowledge (base = <4 correct out of 5) |  |  |  |
| 4 out of 5 correct | 1.01 | 1.05 | 1.50 |
|  | (0.57 - 1.78) | (0.45 - 2.48) | (0.69 - 3.24) |
| All correct | 0.97 | 1.78 | 1.10 |
|  | (0.58 - 1.63) | (0.75 - 4.22) | (0.41 - 2.91) |
| Missing data | 1.03 | 2.11 |  |
|  | (0.21 - 5.06) | (0.23 - 19.24) |  |
| Partner type (base = married/cohabiting) |  |  |  |
| Main partner | 0.39*** | 0.68 | 0.90 |
|  | (0.24 - 0.63) | (0.31 - 1.49) | (0.25 - 3.21) |
| Casual partner | 0.44** | 1.75 | 1.50 |
|  | (0.23 - 0.84) | (0.71 - 4.30) | (0.37 - 6.02) |
| Missing data | 1.29 |  |  |
|  | (0.22 - 7.46) |  |  |
| Partnership length (base = <1 month) |  |  |  |
| 2-6 months | 2.58 | 2.26 | 1.53 |
|  | (0.73 - 9.09) | (0.36 - 14.01) | (0.17 - 14.03) |
| 6-12 months | 3.11** | 2.11 | 1.52 |
|  | (1.06 - 9.07) | (0.44 - 10.16) | (0.21 - 11.13) |
| >1 year | 3.50** | 1.21 | 1.56 |
|  | (1.32 - 9.27) | (0.21 - 6.92) | (0.23 - 10.43) |
| Missing data | 1.65 |  | 3.77 |
|  | (0.31 - 8.83) |  | (0.38 - 36.85) |
| Know partner’s HIV status | 0.87 | 1.95** | 0.84 |
|  | (0.61 - 1.25) | (1.01 - 3.78) | (0.40 - 1.77) |
| Constant | 0.31 | 0.03* | 0.01** |
|  | (0.02 - 3.83) | (0.00 - 1.80) | (0.00 - 0.80) |
|  |  |  |  |
| Observations | 816 | 785 | 780 |

**Notes**: Adjusted odds ratios presented

*** p<0.01, ** p<0.05, * p<0.1

95% Confidence Intervals in parentheses

All analyses are adjusted to account for the complex study design and non-response.
