# Supplementary material for: Sexual Behaviour of Men and Women within Age-Disparate Partnerships in South Africa: Implications for Young Women's HIV Risk
Source: PLoS One. 2016 Aug 15;11(8):e0159162. doi: 10.1371/journal.pone.0159162 (PMC4985138; doi:10.1371/journal.pone.0159162)
Supplement: S2 Table — (DOCX) [file pone.0159162.s002.docx]

**S2 Table.** Full multivariable logistic regression results for the models presented in Table 2, Panel B.

|  | B1 | B2 | B3 |
| --- | --- | --- | --- |
| VARIABLES | Unprotected last sex | Received gifts for sex | Alcohol and sex |
|  |  |  |  |
| Age disparate | 1.44 | 1.20 | 1.02 |
|  | (0.93 - 2.24) | (0.53 - 2.73) | (0.54 - 1.95) |
| Age disparate*rural | 1.12 | 0.99 | 2.40 |
|  | (0.55 - 2.25) | (0.26 - 3.80) | (0.57 - 10.17) |
| Rural | 0.88 | 0.72 | 0.35 |
|  | (0.55 - 1.40) | (0.25 - 2.11) | (0.07 - 1.62) |
| Age (16-24) | 1.06 | 1.09 | 1.06 |
|  | (0.98 - 1.16) | (0.92 - 1.28) | (0.90 - 1.25) |
| Born in South Africa | 0.60 | 0.54 |  |
|  | (0.18 - 2.02) | (0.12 - 2.35) |  |
| Completed Grade 12 | 0.70* | 1.44 | 0.98 |
|  | (0.48 - 1.02) | (0.67 - 3.09) | (0.48 - 2.01) |
| Employed (base = no) |  |  |  |
| Employed | 1.04 | 0.73 | 0.52 |
|  | (0.63 - 1.70) | (0.27 - 2.00) | (0.18 - 1.48) |
| Missing data | 1.60 | 2.54 | 0.13* |
|  | (0.41 - 6.17) | (0.22 - 29.92) | (0.01 - 1.31) |
| Assets (0-7) | 0.90** | 0.89 | 1.20** |
|  | (0.83 - 0.99) | (0.75 - 1.05) | (1.01 - 1.41) |
| HIV tested (base = “no”) |  |  |  |
| Been tested | 1.21 | 0.30*** | 0.57 |
|  | (0.74 - 1.97) | (0.14 - 0.65) | (0.25 - 1.28) |
| Missing data | 2.19 | 4.35 | 0.76 |
|  | (0.40 - 11.93) | (0.68 - 27.70) | (0.06 - 9.49) |
| HIV knowledge (base = <4 correct out of 5) |  |  |  |
| 4 out of 5 correct | 1.00 | 1.05 | 1.46 |
|  | (0.57 - 1.77) | (0.45 - 2.46) | (0.68 - 3.16) |
| All correct | 0.97 | 1.79 | 1.06 |
|  | (0.58 - 1.62) | (0.77 - 4.14) | (0.40 - 2.76) |
| Missing data | 1.02 | 2.11 |  |
|  | (0.20 - 5.12) | (0.23 - 19.16) |  |
| Partner type (base = married/cohabiting) |  |  |  |
| Main partner | 0.39*** | 0.68 | 0.91 |
|  | (0.24 - 0.63) | (0.31 - 1.49) | (0.26 - 3.25) |
| Casual partner | 0.44** | 1.75 | 1.53 |
|  | (0.23 - 0.84) | (0.71 - 4.31) | (0.39 - 6.07) |
| Missing data | 1.26 |  |  |
|  | (0.21 - 7.45) |  |  |
| Partnership length (base = <1 month) |  |  |  |
| 2-6 months | 2.59 | 2.26 | 1.54 |
|  | (0.74 - 9.09) | (0.36 - 14.38) | (0.17 - 13.94) |
| 6-12 months | 3.11** | 2.11 | 1.53 |
|  | (1.07 - 9.07) | (0.44 - 10.17) | (0.21 - 11.11) |
| >1 year | 3.51** | 1.21 | 1.59 |
|  | (1.33 - 9.28) | (0.21 - 7.00) | (0.24 - 10.52) |
| Missing data | 1.64 |  | 3.94 |
|  | (0.31 - 8.81) |  | (0.42 - 37.06) |
| Know partner’s HIV status | 0.87 | 1.95** | 0.84 |
|  | (0.61 - 1.25) | (1.01 - 3.79) | (0.40 - 1.76) |
| Constant | 0.31 | 0.03* | 0.01** |
|  | (0.02 - 3.95) | (0.00 - 1.70) | (0.00 - 0.93) |
|  |  |  |  |
| Observations | 816 | 785 | 780 |

Notes: Adjusted odds ratios presented

*** p<0.01, ** p<0.05, * p<0.1

95% Confidence Intervals in parentheses
