# Supplementary material for: Sexual Behaviour of Men and Women within Age-Disparate Partnerships in South Africa: Implications for Young Women's HIV Risk
Source: PLoS One. 2016 Aug 15;11(8):e0159162. doi: 10.1371/journal.pone.0159162 (PMC4985138; doi:10.1371/journal.pone.0159162)
Supplement: S3 Table — (DOCX) [file pone.0159162.s003.docx]

**S3 Table.** Ordinary Least Squares regression models of sexual behaviours in partnerships reported by 16 to 24 year old women, with the inclusion of the interaction term ‘age-disparate*rural’

|  | B1 | B2 | B3 |
| --- | --- | --- | --- |
| VARIABLES | Unprotected last sex | Received gifts for sex | Alcohol and sex |
|  |  |  |  |
| Age disparate (vs similar-aged) | 0.08 | 0.01 | 0.00 |
|  | (-0.02 - 0.18) | (-0.04 - 0.06) | (-0.04 - 0.05) |
| Age disparate*rural | 0.03 | -0.00 | 0.03 |
|  | (-0.13 - 0.19) | (-0.08 - 0.07) | (-0.03 - 0.09) |
| Rural | -0.03 | -0.02 | -0.04 |
|  | (-0.13 - 0.07) | (-0.08 - 0.04) | (-0.09 - 0.01) |
| Age (16-24) | 0.01 | 0.00 | 0.00 |
|  | (-0.00 - 0.03) | (-0.01 - 0.01) | (-0.01 - 0.01) |
| Born in South Africa | -0.10 | -0.04 | 0.06** |
|  | (-0.34 - 0.13) | (-0.15 - 0.08) | (0.01 - 0.11) |
| Completed Grade 12 | -0.08* | 0.02 | -0.00 |
|  | (-0.17 - 0.00) | (-0.02 - 0.07) | (-0.04 - 0.04) |
| Employed (base = no) |  |  |  |
| Employed | 0.01 | -0.02 | -0.03 |
|  | (-0.10 - 0.12) | (-0.07 - 0.03) | (-0.08 - 0.01) |
| Missing data | 0.10 | 0.06 | -0.08* |
|  | (-0.19 - 0.39) | (-0.15 - 0.26) | (-0.15 - 0.00) |
| Assets (0-7) | -0.02** | -0.01 | 0.01** |
|  | (-0.04 - -0.00) | (-0.02 - 0.00) | (0.00 - 0.02) |
| HIV tested (base = “no”) |  |  |  |
| Been tested | 0.04 | -0.08** | -0.03 |
|  | (-0.06 - 0.15) | (-0.14 - -0.02) | (-0.09 - 0.02) |
| Missing data | 0.18 | 0.21 | -0.03 |
|  | (-0.21 - 0.58) | (-0.16 - 0.57) | (-0.14 - 0.09) |
| HIV knowledge (base = <4 correct out of 5) |  |  |  |
| 4 out of 5 correct | 0.00 | 0.00 | 0.02 |
|  | (-0.13 - 0.13) | (-0.04 - 0.05) | (-0.02 - 0.07) |
| All correct | -0.01 | 0.03 | 0.00 |
|  | (-0.12 - 0.11) | (-0.02 - 0.08) | (-0.05 - 0.05) |
| Missing data | 0.00 | 0.03 | -0.07** |
|  | (-0.37 - 0.38) | (-0.11 - 0.18) | (-0.12 - -0.01) |
| Partner type (base = married/cohabiting) |  |  |  |
| Main partner | -0.22*** | -0.02 | -0.00 |
|  | (-0.33 - -0.12) | (-0.07 - 0.03) | (-0.06 - 0.06) |
| Casual partner | -0.20*** | 0.04 | 0.04 |
|  | (-0.34 - -0.05) | (-0.03 - 0.12) | (-0.04 - 0.12) |
| Missing data | 0.01 | -0.08 | -0.01 |
|  | (-0.32 - 0.34) | (-0.21 - 0.06) | (-0.12 - 0.09) |
| Partnership length (base = <1 month) |  |  |  |
| 2-6 months | 0.18 | 0.05 | 0.03 |
|  | (-0.05 - 0.40) | (-0.07 - 0.17) | (-0.08 - 0.14) |
| 6-12 months | 0.22** | 0.04 | 0.03 |
|  | (0.04 - 0.40) | (-0.04 - 0.12) | (-0.07 - 0.13) |
| >1 year | 0.25*** | 0.00 | 0.03 |
|  | (0.09 - 0.40) | (-0.08 - 0.09) | (-0.07 - 0.12) |
| Missing data | 0.10 | -0.06 | 0.13 |
|  | (-0.18 - 0.37) | (-0.14 - 0.02) | (-0.08 - 0.33) |
| Know partner’s HIV status | -0.03 | 0.03* | -0.01 |
|  | (-0.11 - 0.05) | (-0.00 - 0.07) | (-0.05 - 0.03) |
| Constant | 0.26 | 0.07 | -0.09 |
|  | (-0.26 - 0.78) | (-0.15 - 0.29) | (-0.32 - 0.13) |
|  |  |  |  |
| Observations | 816 | 813 | 815 |

**Notes**: Adjusted odds ratios presented

*** p<0.01, ** p<0.05, * p<0.1

95% Confidence Intervals in parentheses

All analyses are adjusted to account for the complex study design and non-response.
