# Supplementary material for: Sexual Behaviour of Men and Women within Age-Disparate Partnerships in South Africa: Implications for Young Women's HIV Risk
Source: PLoS One. 2016 Aug 15;11(8):e0159162. doi: 10.1371/journal.pone.0159162 (PMC4985138; doi:10.1371/journal.pone.0159162)
Supplement: S4 Table — (DOCX) [file pone.0159162.s004.docx]

**S4 Table.** Full multivariable logistic regression results for the models presented in Table 3, Panel A.

|  | 1 | 2 | 3 | 4 |
| --- | --- | --- | --- | --- |
| VARIABLES | Unprotected last sex | Gave gifts for sex | Alcohol and sex | Concurrency |
|  |  |  |  |  |
| Age disparate (vs similar-aged) | 1.92*** | 2.73*** | 1.60* | 1.39* |
|  | (1.31 - 2.81) | (1.64 - 4.56) | (0.98 - 2.61) | (0.94 - 2.07) |
| Rural | 1.03 | 0.90 | 0.51** | 0.52** |
|  | (0.73 - 1.48) | (0.47 - 1.73) | (0.29 - 0.89) | (0.31 - 0.86) |
| Female partner’s age (16-24) | 1.08** | 1.04 | 0.98 | 1.07 |
|  | (1.02 - 1.15) | (0.93 - 1.17) | (0.89 - 1.08) | (0.98 - 1.17) |
| Born in South Africa | 1.07 | 0.40** | 0.71 | 1.00 |
|  | (0.54 - 2.15) | (0.17 - 0.97) | (0.32 - 1.60) | (0.46 - 2.17) |
| Completed Grade 12 | 0.60** | 1.27 | 0.89 | 1.23 |
|  | (0.41 - 0.89) | (0.65 - 2.47) | (0.55 - 1.43) | (0.76 - 1.99) |
| Employed (base = no) |  |  |  |  |
| Employed | 1.10 | 1.46 | 1.16 | 1.68** |
|  | (0.78 - 1.54) | (0.77 - 2.77) | (0.65 - 2.05) | (1.02 - 2.78) |
| Missing data | 0.88 |  |  | 0.54 |
|  | (0.14 - 5.44) |  |  | (0.08 - 3.70) |
| Assets (0-7) | 0.83*** | 0.98 | 1.10 | 0.96 |
|  | (0.77 - 0.91) | (0.86 - 1.13) | (0.97 - 1.24) | (0.86 - 1.08) |
| HIV tested (base = “no”) |  |  |  |  |
| Been tested | 0.95 | 0.86 | 1.13 | 0.80 |
|  | (0.63 - 1.41) | (0.51 - 1.46) | (0.75 - 1.70) | (0.47 - 1.35) |
| Missing data | 5.59*** | 3.02 | 1.86 | 1.86 |
|  | (1.85 - 16.91) | (0.77 - 11.81) | (0.49 - 7.09) | (0.47 - 7.37) |
| HIV knowledge (base = <4 correct out of 5) |  |  |  |  |
| 4 out of 5 correct | 0.66* | 0.57 | 1.02 | 1.13 |
|  | (0.41 - 1.06) | (0.26 - 1.23) | (0.54 - 1.94) | (0.63 - 2.03) |
| All correct | 0.80 | 1.33 | 0.85 | 1.29 |
|  | (0.49 - 1.31) | (0.63 - 2.82) | (0.44 - 1.63) | (0.70 - 2.36) |
| Missing data | 3.77** | 0.60 | 1.05 | 0.25 |
|  | (1.14 - 12.41) | (0.08 - 4.32) | (0.17 - 6.39) | (0.02 - 2.98) |
| Partner type (base = married/cohabiting) |  |  |  |  |
| Main partner | 0.36*** | 1.86 | 0.55* | 1.48 |
|  | (0.21 - 0.61) | (0.68 - 5.10) | (0.29 - 1.05) | (0.71 - 3.08) |
| Casual partner | 0.24*** | 3.47** | 1.02 | 9.60*** |
|  | (0.12 - 0.45) | (1.24 - 9.71) | (0.55 - 1.89) | (4.15 - 22.22) |
| Missing data | 0.36 | 5.91 | 2.99 | 26.76*** |
|  | (0.05 - 2.85) | (0.45 - 77.54) | (0.35 - 25.6) | (3.63 - 197.4) |
| Partnership length (base = <1 month) |  |  |  |  |
| 2-6 months | 1.56 | 0.84 | 0.45** | 0.97 |
|  | (0.81 - 3.00) | (0.41 - 1.69) | (0.20 - 0.98) | (0.51 - 1.84) |
| 6-12 months | 1.16 | 0.43** | 0.32*** | 0.62* |
|  | (0.58 - 2.32) | (0.21 - 0.88) | (0.16 - 0.64) | (0.37 - 1.04) |
| >1 year | 2.04** | 0.42** | 0.53** | 0.49*** |
|  | (1.17 - 3.57) | (0.19 - 0.92) | (0.33 - 0.86) | (0.29 - 0.80) |
| Missing data | 2.46* | 1.67 | 0.25** | 0.36** |
|  | (1.00 - 6.06) | (0.64 - 4.36) | (0.06 - 0.94) | (0.13 - 0.98) |
| Know partner’s HIV status | 0.78 | 0.61 | 0.77 | 0.64* |
|  | (0.52 - 1.16) | (0.31 - 1.20) | (0.48 - 1.24) | (0.40 - 1.03) |
| Constant | 0.46 | 0.06** | 0.87 | 0.06** |
|  | (0.08 - 2.61) | (0.00 - 0.96) | (0.12 - 6.33) | (0.01 - 0.52) |
|  |  |  |  |  |
| Observations | 980 | 961 | 966 | 982 |

**Notes**: Adjusted odds ratios presented

*** p<0.01, ** p<0.05, * p<0.1

95% Confidence Intervals in parentheses

All analyses are adjusted to account for the complex study design and non-response.
