# Supplementary material for: Sexual Behaviour of Men and Women within Age-Disparate Partnerships in South Africa: Implications for Young Women's HIV Risk
Source: PLoS One. 2016 Aug 15;11(8):e0159162. doi: 10.1371/journal.pone.0159162 (PMC4985138; doi:10.1371/journal.pone.0159162)
Supplement: S5 Table — (DOCX) [file pone.0159162.s005.docx]

**S5 Table.** Full multivariable logistic regression results for the models presented in Table 3, Panel B.

|  | 1B | 2B | 3B | 4B |
| --- | --- | --- | --- | --- |
| VARIABLES | Unprotected last sex | Gave gifts for sex | Alcohol and sex | Concurrency |
|  |  |  |  |  |
| Age disparate | 1.70*** | 4.28*** | 2.38*** | 1.28 |
|  | (1.15 - 2.52) | (2.25 - 8.13) | (1.28 - 4.46) | (0.80 - 2.07) |
| Age disparate*rural | 1.29 | 0.34** | 0.27** | 1.22 |
|  | (0.55 - 3.05) | (0.12 - 0.92) | (0.10 - 0.74) | (0.54 - 2.79) |
| Rural | 0.94 | 1.55 | 0.84 | 0.48** |
|  | (0.60 - 1.48) | (0.67 - 3.60) | (0.46 - 1.53) | (0.25 - 0.90) |
| Female partner’s age (16-24) | 1.08** | 1.06 | 0.99 | 1.07 |
|  | (1.01 - 1.15) | (0.95 - 1.20) | (0.90 - 1.09) | (0.98 - 1.16) |
| Born in South Africa | 1.07 | 0.39** | 0.72 | 0.99 |
|  | (0.54 - 2.12) | (0.16 - 0.96) | (0.33 - 1.61) | (0.47 - 2.12) |
| Completed Grade 12 | 0.60** | 1.32 | 0.94 | 1.22 |
|  | (0.40 - 0.90) | (0.68 - 2.56) | (0.57 - 1.54) | (0.76 - 1.98) |
| Employed (base = no) |  |  |  |  |
| Employed | 1.11 | 1.39 | 1.12 | 1.69** |
|  | (0.78 - 1.57) | (0.72 - 2.69) | (0.63 - 1.98) | (1.02 - 2.81) |
| Missing data | 0.89 |  |  | 0.55 |
|  | (0.15 - 5.25) |  |  | (0.08 - 3.81) |
| Assets (0-7) | 0.83*** | 0.98 | 1.09 | 0.96 |
|  | (0.77 - 0.91) | (0.85 - 1.13) | (0.97 - 1.23) | (0.86 - 1.08) |
| HIV tested (base = “no”) |  |  |  |  |
| Been tested | 0.94 | 0.87 | 1.17 | 0.79 |
|  | (0.63 - 1.41) | (0.51 - 1.49) | (0.78 - 1.75) | (0.47 - 1.34) |
| Missing data | 5.69*** | 3.01 | 1.88 | 1.86 |
|  | (1.84 - 17.58) | (0.77 - 11.74) | (0.46 - 7.76) | (0.48 - 7.30) |
| HIV knowledge (base = <4 correct out of 5) |  |  |  |  |
| 4 out of 5 correct | 0.66* | 0.57 | 1.06 | 1.14 |
|  | (0.41 - 1.06) | (0.27 - 1.22) | (0.56 - 2.01) | (0.64 - 2.03) |
| All correct | 0.80 | 1.37 | 0.89 | 1.28 |
|  | (0.49 - 1.30) | (0.66 - 2.86) | (0.47 - 1.70) | (0.70 - 2.35) |
| Missing data | 3.82** | 0.59 | 1.00 | 0.24 |
|  | (1.18 - 12.39) | (0.07 - 4.73) | (0.16 - 6.05) | (0.02 - 2.99) |
| Partner type (base = married/cohabiting) |  |  |  |  |
| Main partner | 0.36*** | 2.07 | 0.59 | 1.45 |
|  | (0.21 - 0.60) | (0.75 - 5.70) | (0.31 - 1.14) | (0.68 - 3.08) |
| Casual partner | 0.23*** | 3.82** | 1.10 | 9.44*** |
|  | (0.12 - 0.43) | (1.36 - 10.79) | (0.58 - 2.08) | (4.03 - 22.11) |
| Missing data | 0.36 | 7.00 | 3.04 | 26.94*** |
|  | (0.05 - 2.76) | (0.54 - 90.87) | (0.36 - 25.65) | (3.56 - 203.55) |
| Partnership length (base = <1 month) |  |  |  |  |
| 2-6 months | 1.55 | 0.86 | 0.46** | 0.96 |
|  | (0.81 - 2.95) | (0.42 - 1.75) | (0.21 - 0.99) | (0.51 - 1.82) |
| 6-12 months | 1.14 | 0.44** | 0.33*** | 0.61* |
|  | (0.57 - 2.25) | (0.21 - 0.92) | (0.17 - 0.66) | (0.36 - 1.03) |
| >1 year | 2.01** | 0.44** | 0.56** | 0.48*** |
|  | (1.16 - 3.46) | (0.20 - 0.99) | (0.35 - 0.91) | (0.29 - 0.80) |
| Missing data | 2.41* | 1.86 | 0.26* | 0.36** |
|  | (0.97 - 5.99) | (0.70 - 4.94) | (0.07 - 1.06) | (0.13 - 0.98) |
| Know partner’s HIV status | 0.78 | 0.60 | 0.77 | 0.64* |
|  | (0.52 - 1.16) | (0.30 - 1.20) | (0.47 - 1.26) | (0.40 - 1.03) |
| Constant | 0.55 | 0.03** | 0.47 | 0.07** |
|  | (0.11 - 2.78) | (0.00 - 0.53) | (0.06 - 3.99) | (0.01 - 0.66) |
|  |  |  |  |  |
| Observations | 980 | 961 | 966 | 982 |

Notes: Adjusted odds ratios presented

*** p<0.01, ** p<0.05, * p<0.1

95% Confidence Intervals in parentheses
