# Supplementary material for: Sexual Behaviour of Men and Women within Age-Disparate Partnerships in South Africa: Implications for Young Women's HIV Risk
Source: PLoS One. 2016 Aug 15;11(8):e0159162. doi: 10.1371/journal.pone.0159162 (PMC4985138; doi:10.1371/journal.pone.0159162)
Supplement: S6 Table — (DOCX) [file pone.0159162.s006.docx]

**S6 Table.** Ordinary Least Squares regression models of sexual behaviours reported by men in partnerships with 16 to 24 year old women, with the inclusion of the interaction term ‘age-disparate*rural’

|  | 1 | 2 | 3 | 4 |
| --- | --- | --- | --- | --- |
| VARIABLES | Unprotected last sex | Gave gifts for sex | Alcohol and sex | Concurrency |
|  |  |  |  |  |
| Age disparate (vs similar-aged) | 0.11*** | 0.13*** | 0.15*** | 0.05 |
|  | (0.03 - 0.18) | (0.07 - 0.20) | (0.04 - 0.26) | (-0.03 - 0.13) |
| Age disparate*rural | 0.07 | -0.11*** | -0.19*** | 0.01 |
|  | (-0.11 - 0.24) | (-0.20 - -0.03) | (-0.31 - -0.06) | (-0.12 - 0.13) |
| Rural | -0.02 | 0.03 | -0.02 | -0.10** |
|  | (-0.10 - 0.07) | (-0.02 - 0.08) | (-0.08 - 0.05) | (-0.19 - -0.01) |
| Female partner’s age (16-24) | 0.02** | 0.00 | -0.00 | 0.01 |
|  | (0.00 - 0.03) | (-0.01 - 0.01) | (-0.01 - 0.01) | (-0.00 - 0.02) |
| Born in South Africa | 0.01 | -0.11* | -0.06 | -0.01 |
|  | (-0.12 - 0.15) | (-0.24 - 0.01) | (-0.22 - 0.09) | (-0.14 - 0.12) |
| Completed Grade 12 | -0.10** | 0.02 | -0.01 | 0.03 |
|  | (-0.19 - -0.02) | (-0.04 - 0.07) | (-0.08 - 0.05) | (-0.05 - 0.10) |
| Employed (base = no) |  |  |  |  |
| Employed | 0.02 | 0.03 | 0.02 | 0.08* |
|  | (-0.05 - 0.09) | (-0.02 - 0.09) | (-0.06 - 0.09) | (-0.00 - 0.16) |
| Missing data | -0.02 | -0.08** | -0.14** | -0.06 |
|  | (-0.43 - 0.39) | (-0.15 - -0.00) | (-0.26 - -0.02) | (-0.24 - 0.12) |
| Assets (0-7) | -0.04*** | 0.00 | 0.01 | -0.01 |
|  | (-0.05 - -0.02) | (-0.01 - 0.01) | (-0.00 - 0.03) | (-0.02 - 0.01) |
| HIV tested (base = “no”) |  |  |  |  |
| Been tested | -0.01 | -0.00 | 0.02 | -0.03 |
|  | (-0.09 - 0.07) | (-0.05 - 0.04) | (-0.03 - 0.07) | (-0.11 - 0.05) |
| Missing data | 0.36*** | 0.21 | 0.13 | 0.12 |
|  | (0.15 - 0.58) | (-0.08 - 0.49) | (-0.16 - 0.42) | (-0.10 - 0.34) |
| HIV knowledge (base = <4 correct out of 5) |  |  |  |  |
| 4 out of 5 correct | -0.08* | -0.04 | 0.01 | 0.02 |
|  | (-0.18 - 0.01) | (-0.10 - 0.02) | (-0.08 - 0.10) | (-0.07 - 0.11) |
| All correct | -0.05 | 0.03 | -0.01 | 0.04 |
|  | (-0.15 - 0.05) | (-0.04 - 0.10) | (-0.09 - 0.08) | (-0.06 - 0.13) |
| Missing data | 0.28** | -0.06 | 0.01 | -0.13 |
|  | (0.05 - 0.51) | (-0.20 - 0.08) | (-0.25 - 0.26) | (-0.31 - 0.05) |
| Partner type (base = married/cohabiting) |  |  |  |  |
| Main partner | -0.23*** | 0.06** | -0.06 | 0.06 |
|  | (-0.33 - -0.12) | (0.00 - 0.12) | (-0.16 - 0.04) | (-0.03 - 0.15) |
| Casual partner | -0.31*** | 0.12*** | 0.01 | 0.42*** |
|  | (-0.43 - -0.19) | (0.04 - 0.19) | (-0.09 - 0.12) | (0.30 - 0.55) |
| Missing data | -0.24 | 0.15 | 0.21 | 0.61*** |
|  | (-0.66 - 0.19) | (-0.17 - 0.47) | (-0.23 - 0.65) | (0.25 - 0.98) |
| Partnership length (base = <1 month) |  |  |  |  |
| 2-6 months | 0.07 | -0.03 | -0.13** | -0.01 |
|  | (-0.04 - 0.19) | (-0.12 - 0.06) | (-0.26 - -0.01) | (-0.14 - 0.11) |
| 6-12 months | 0.02 | -0.09** | -0.17*** | -0.11** |
|  | (-0.10 - 0.13) | (-0.17 - -0.00) | (-0.28 - -0.07) | (-0.21 - -0.00) |
| >1 year | 0.13*** | -0.08* | -0.11** | -0.14*** |
|  | (0.03 - 0.22) | (-0.17 - 0.00) | (-0.21 - -0.02) | (-0.24 - -0.04) |
| Missing data | 0.17* | 0.07 | -0.19*** | -0.18** |
|  | (-0.01 - 0.34) | (-0.09 - 0.23) | (-0.33 - -0.05) | (-0.34 - -0.01) |
| Know partner’s HIV status | -0.05 | -0.04* | -0.03 | -0.07* |
|  | (-0.13 - 0.03) | (-0.09 - 0.00) | (-0.09 - 0.03) | (-0.14 - 0.00) |
| Constant | 0.39** | 0.06 | 0.34** | 0.04 |
|  | (0.07 - 0.71) | (-0.22 - 0.34) | (0.03 - 0.65) | (-0.30 - 0.39) |
|  |  |  |  |  |
| Observations | 980 | 966 | 971 | 982 |

**Notes**: *** p<0.01, ** p<0.05, * p<0.1

95% Confidence Intervals in parentheses

All analyses are adjusted to account for the complex study design and non-response.
