# Supplementary material for: Sexual Behaviour of Men and Women within Age-Disparate Partnerships in South Africa: Implications for Young Women's HIV Risk
Source: PLoS One. 2016 Aug 15;11(8):e0159162. doi: 10.1371/journal.pone.0159162 (PMC4985138; doi:10.1371/journal.pone.0159162)
Supplement: S7 Table — (DOCX) [file pone.0159162.s007.docx]

**S7 Table**. Multivariable logistic regression models of transactional sex and alcohol consumption *within* rural and urban areas as reported by men in partnership with 16-24 year old women.

|  | **Rural** | | **Urban** | |
| --- | --- | --- | --- | --- |
| VARIABLES | Gave gifts for sex | Alcohol and sex | Gave gifts for sex | Alcohol and sex |
|  |  |  |  |  |
| Age disparate (vs similar-aged) | 1.04 | 0.79 | 4.14*** | 2.24** |
|  | (0.42 - 2.58) | (0.35 - 1.81) | (2.03 - 8.46) | (1.20 - 4.19) |
| Female partner’s age (16-24) | 1.10 | 1.01 | 1.01 | 0.97 |
|  | (0.87 - 1.38) | (0.80 - 1.28) | (0.88 - 1.16) | (0.88 - 1.07) |
| Born in South Africa | - | - | 0.28*** | 0.75 |
|  |  |  | (0.12 - 0.67) | (0.33 - 1.72) |
| Completed Grade 12 | 2.00 | 0.96 | 1.03 | 0.87 |
|  | (0.73 - 5.50) | (0.40 - 2.28) | (0.46 - 2.29) | (0.48 - 1.56) |
| Employed (base = no) |  |  |  |  |
| Employed | 2.06 | 0.77 | 1.22 | 1.50 |
|  | (0.58 - 7.32) | (0.16 - 3.65) | (0.59 - 2.53) | (0.86 - 2.62) |
| Missing data |  |  |  |  |
|  |  |  |  |  |
| Assets (0-7) | 0.94 | 1.17 | 1.01 | 1.05 |
|  | (0.72 - 1.23) | (0.90 - 1.52) | (0.85 - 1.19) | (0.92 - 1.21) |
| HIV tested (base = “no”) |  |  |  |  |
| Been tested | 1.17 | 0.95 | 0.75 | 1.27 |
|  | (0.48 - 2.85) | (0.47 - 1.91) | (0.41 - 1.35) | (0.77 - 2.10) |
| Missing data |  | 2.02 | 4.63** | 1.67 |
|  |  | (0.18 - 23.16) | (1.04 - 20.61) | (0.30 - 9.29) |
| HIV knowledge (base = <4 correct out of 5) |  |  |  |  |
| 4 out of 5 correct | 0.34 | 0.65 | 0.73 | 1.32 |
|  | (0.09 - 1.25) | (0.22 - 1.98) | (0.28 - 1.87) | (0.63 - 2.79) |
| All correct | 0.75 | 0.19*** | 1.74 | 1.67 |
|  | (0.23 - 2.41) | (0.06 - 0.58) | (0.68 - 4.49) | (0.75 - 3.73) |
| Missing data | 0.92 | 0.69 |  | 0.99 |
|  | (0.07 - 13.07) | (0.06 - 8.40) |  | (0.10 - 9.36) |
| Partner type (base = married/cohabiting) |  |  |  |  |
| Main partner | 1.41 | 1.10 | 2.53* | 0.53 |
|  | (0.32 - 6.15) | (0.24 - 4.97) | (0.85 - 7.54) | (0.23 - 1.21) |
| Casual partner | 1.65 | 1.91 | 6.04*** | 0.97 |
|  | (0.30 - 9.02) | (0.39 - 9.43) | (2.53 - 14.42) | (0.46 - 2.05) |
| Missing data |  | 2.59 | 14.91* | 2.44 |
|  |  | (0.05 - 135.93) | (0.70 - 318.52) | (0.30 - 19.91) |
| Partnership length (base = <1 month) |  |  |  |  |
| 2-6 months | 0.11** | 0.53 | 0.83 | 0.43** |
|  | (0.02 - 0.71) | (0.07 - 4.29) | (0.36 - 1.93) | (0.19 - 0.96) |
| 6-12 months | 0.07*** | 0.39 | 0.40** | 0.30*** |
|  | (0.01 - 0.44) | (0.06 - 2.45) | (0.18 - 0.88) | (0.14 - 0.63) |
| >1 year | 0.14** | 0.59 | 0.23*** | 0.52*** |
|  | (0.03 - 0.72) | (0.14 - 2.51) | (0.08 - 0.66) | (0.32 - 0.84) |
| Missing data |  | 0.72 | 0.63 | 0.15* |
|  |  | (0.10 - 5.32) | (0.16 - 2.51) | (0.02 - 1.00) |
| Know partner’s HIV status | 0.92 | 1.09 | 0.43* | 0.68 |
|  | (0.32 - 2.68) | (0.37 - 3.19) | (0.19 - 1.00) | (0.40 - 1.16) |
| Constant | 0.06 | 0.16 | 0.11 | 0.73 |
|  | (0.00 - 9.45) | (0.00 - 33.00) | (0.00 - 2.73) | (0.09 - 6.10) |
|  |  |  |  |  |
| Observations | 294 | 326 | 629 | 638 |

**Notes**: Adjusted odds ratios presented

*** p<0.01, ** p<0.05, * p<0.1

95% Confidence Intervals in parentheses

All analyses are adjusted to account for the complex study design and non-response.
