# Supplementary material for: Sexual Behaviour of Men and Women within Age-Disparate Partnerships in South Africa: Implications for Young Women's HIV Risk
Source: PLoS One. 2016 Aug 15;11(8):e0159162. doi: 10.1371/journal.pone.0159162 (PMC4985138; doi:10.1371/journal.pone.0159162)
Supplement: S8 Table — (DOCX) [file pone.0159162.s008.docx]

**S8 Table.** Multivariable logistic regression models of inconsistent condom use within partnerships reported by 16 to 24 year old women.

In the model presented here, the dependent variable ‘inconsistent condom use’ identifies partnerships in which condoms were reported to be used “never”, “hardly ever” or “some of the time.”

| VARIABLES | Inconsistent condom use |
| --- | --- |
|  |  |
| Age disparate (vs similar-aged) | 1.42** |
|  | (1.03 - 1.98) |
| Age disparate*rural | na |
|  | na |
| Rural | 1.14 |
|  | (0.78 - 1.67) |
| Age (16-24) | 1.05 |
|  | (0.97 - 1.14) |
| Born in South Africa | 0.30 |
|  | (0.05 - 1.70) |
| Completed Grade 12 | 0.68** |
|  | (0.47 - 0.98) |
| Employed (base = no) |  |
| Employed | 1.04 |
|  | (0.61 - 1.77) |
| Missing data | 1.25 |
|  | (0.29 - 5.35) |
| Assets (0-7) | 0.81*** |
|  | (0.74 - 0.89) |
| HIV tested (base = “no”) |  |
| Been tested | 1.56* |
|  | (0.94 - 2.57) |
| Missing data | 1.41 |
|  | (0.14 - 13.85) |
| HIV knowledge (base = <4 correct out of 5) |  |
| 4 out of 5 correct | 0.80 |
|  | (0.46 - 1.40) |
| All correct | 0.71 |
|  | (0.43 - 1.19) |
| Missing data | 3.03 |
|  | (0.65 - 14.22) |
| Partner type (base = married/cohabiting) |  |
| Main partner | 0.34*** |
|  | (0.18 - 0.62) |
| Casual partner | 0.30*** |
|  | (0.13 - 0.69) |
| Missing data | 0.52 |
|  | (0.09 - 2.96) |
| Partnership length (base = <1 month) |  |
| 2-6 months | 1.28 |
|  | (0.47 - 3.51) |
| 6-12 months | 2.06 |
|  | (0.83 - 5.15) |
| >1 year | 2.03* |
|  | (0.88 - 4.66) |
| Missing data | 0.91 |
|  | (0.19 - 4.30) |
| Know partner’s HIV status | 1.09 |
|  | (0.73 - 1.63) |
| Constant | 3.57 |
|  | (0.22 - 57.78) |
|  |  |
| Observations | 818 |

**Notes**: Adjusted odds ratios presented

*** p<0.01, ** p<0.05, * p<0.1

95% Confidence Intervals in parentheses

All analyses are adjusted to account for the complex study design and non-response.
