# Supplementary material for: Sexual Behaviour of Men and Women within Age-Disparate Partnerships in South Africa: Implications for Young Women's HIV Risk
Source: PLoS One. 2016 Aug 15;11(8):e0159162. doi: 10.1371/journal.pone.0159162 (PMC4985138; doi:10.1371/journal.pone.0159162)
Supplement: S10 Table — (DOCX) [file pone.0159162.s010.docx]

**S10 Table.** Multivariable logistic regression models of sexual behaviours within partnerships reported by 16 to 24 year old women (independent variable = years age difference).

In the models presented here, the independent variable of interested, ‘age difference in years’ was created as the difference in age between the respondent and his/her partner. In all models the independent variable represents the number of years the male partner in the partnerships is older than the female partner.

|  | 1 | 2 | 3 |
| --- | --- | --- | --- |
| VARIABLES | Unprotected last sex | Received gifts for sex | Alcohol and sex |
|  |  |  |  |
| Years age difference | 1.09*** | 1.05 | 1.05 |
|  | (1.04 - 1.15) | (0.96 - 1.15) | (0.98 - 1.12) |
| Rural | 0.95 | 0.74 | 0.56 |
|  | (0.64 - 1.42) | (0.31 - 1.77) | (0.19 - 1.60) |
| Age (16-24) | 1.07 | 1.09 | 1.06 |
|  | (0.98 - 1.17) | (0.93 - 1.28) | (0.90 - 1.25) |
| Born in South Africa | 0.55 | 0.49 |  |
|  | (0.16 - 1.85) | (0.11 - 2.18) |  |
| Completed Grade 12 | 0.70* | 1.45 | 0.97 |
|  | (0.47 - 1.02) | (0.68 - 3.11) | (0.47 - 2.01) |
| Employed (base = no) |  |  |  |
| Employed | 1.01 | 0.73 | 0.51 |
|  | (0.61 - 1.67) | (0.27 - 2.00) | (0.18 - 1.43) |
| Missing data | 1.51 | 2.41 | 0.12* |
|  | (0.40 - 5.71) | (0.20 - 28.41) | (0.01 - 1.32) |
| Assets (0-7) | 0.91** | 0.89 | 1.21** |
|  | (0.83 - 0.99) | (0.75 - 1.05) | (1.02 - 1.43) |
| HIV tested (base = “no”) |  |  |  |
| Been tested | 1.21 | 0.30*** | 0.58 |
|  | (0.74 - 1.97) | (0.14 - 0.65) | (0.26 - 1.30) |
| Missing data | 2.33 | 4.66 | 0.78 |
|  | (0.42 - 12.81) | (0.73 - 29.61) | (0.06 - 10.22) |
| HIV knowledge (base = <4 correct out of 5) |  |  |  |
| 4 out of 5 correct | 1.08 | 1.12 | 1.60 |
|  | (0.61 - 1.91) | (0.48 - 2.60) | (0.74 - 3.43) |
| All correct | 1.05 | 1.92 | 1.17 |
|  | (0.62 - 1.76) | (0.79 - 4.66) | (0.44 - 3.12) |
| Missing data | 1.15 | 2.27 |  |
|  | (0.24 - 5.51) | (0.24 - 21.35) |  |
| Partner type (base = married/cohabiting) |  |  |  |
| Main partner | 0.41*** | 0.71 | 0.93 |
|  | (0.26 - 0.66) | (0.32 - 1.57) | (0.26 - 3.27) |
| Casual partner | 0.46** | 1.84 | 1.53 |
|  | (0.24 - 0.90) | (0.74 - 4.58) | (0.38 - 6.12) |
| Missing data | 1.51 |  |  |
|  | (0.25 - 9.18) |  |  |
| Partnership length (base = <1 month) |  |  |  |
| 2-6 months | 2.76 | 2.37 | 1.50 |
|  | (0.74 - 10.28) | (0.37 - 15.21) | (0.17 - 13.52) |
| 6-12 months | 3.42** | 2.23 | 1.53 |
|  | (1.10 - 10.60) | (0.45 - 11.11) | (0.21 - 11.06) |
| >1 year | 3.82** | 1.27 | 1.55 |
|  | (1.35 - 10.80) | (0.21 - 7.51) | (0.24 - 10.26) |
| Missing data | 1.81 |  | 3.79 |
|  | (0.34 - 9.74) |  | (0.39 - 36.85) |
| Know partner’s HIV status | 0.91 | 2.01** | 0.85 |
|  | (0.63 - 1.30) | (1.02 - 3.98) | (0.41 - 1.78) |
| Constant | 0.19 | 0.02* | 0.01** |
|  | (0.01 - 2.53) | (0.00 - 1.19) | (0.00 - 0.75) |
|  |  |  |  |
| Observations | 816 | 785 | 780 |

**Notes**: Adjusted odds ratios presented

*** p<0.01, ** p<0.05, * p<0.1

95% Confidence Intervals in parentheses

All analyses are adjusted to account for the complex study design and non-response.
