# Supplementary material for: Sexual Behaviour of Men and Women within Age-Disparate Partnerships in South Africa: Implications for Young Women's HIV Risk
Source: PLoS One. 2016 Aug 15;11(8):e0159162. doi: 10.1371/journal.pone.0159162 (PMC4985138; doi:10.1371/journal.pone.0159162)
Supplement: S11 Table — (DOCX) [file pone.0159162.s011.docx]

**S11 Table**. Multivariable logistic regression models of sexual behaviours reported by men in partnerships with 16 to 24 year old women (independent variable = years age difference).

In the models presented here, the independent variable of interested, ‘age difference in years’ was created as the difference in age between the respondent and his/her partner. In all models the independent variable represents the number of years the male partner in the partnerships is older than the female partner.

|  | 1 | 2 | 3 | 4 |
| --- | --- | --- | --- | --- |
| VARIABLES | Unprotected last sex | Gave gifts for sex | Alcohol and sex | Concurrency |
|  |  |  |  |  |
| Years age difference | 1.06** | 1.14*** | 1.08** | 1.05* |
|  | (1.01 - 1.10) | (1.08 - 1.21) | (1.01 - 1.14) | (0.99 - 1.11) |
| Rural | 1.03 | 0.97 | 0.53** | 0.53** |
|  | (0.72 - 1.48) | (0.50 - 1.90) | (0.31 - 0.92) | (0.32 - 0.88) |
| Female partner’s age (16-24) | 1.08** | 1.04 | 0.98 | 1.07 |
|  | (1.02 - 1.15) | (0.93 - 1.16) | (0.89 - 1.08) | (0.98 - 1.17) |
| Born in South Africa | 1.02 | 0.36** | 0.70 | 0.99 |
|  | (0.52 - 1.98) | (0.15 - 0.87) | (0.31 - 1.58) | (0.45 - 2.18) |
| Completed Grade 12 | 0.60** | 1.35 | 0.92 | 1.25 |
|  | (0.41 - 0.89) | (0.71 - 2.56) | (0.57 - 1.48) | (0.78 - 2.01) |
| Employed (base = no) |  |  |  |  |
| Employed | 1.10 | 1.30 | 1.07 | 1.63* |
|  | (0.78 - 1.56) | (0.66 - 2.53) | (0.60 - 1.92) | (0.98 - 2.71) |
| Missing data | 0.77 |  |  | 0.42 |
|  | (0.12 - 4.95) |  |  | (0.07 - 2.61) |
| Assets (0-7) | 0.83*** | 0.98 | 1.10 | 0.96 |
|  | (0.77 - 0.91) | (0.85 - 1.13) | (0.97 - 1.24) | (0.86 - 1.08) |
| HIV tested (base = “no”) |  |  |  |  |
| Been tested | 0.92 | 0.80 | 1.11 | 0.79 |
|  | (0.62 - 1.38) | (0.46 - 1.38) | (0.73 - 1.69) | (0.46 - 1.34) |
| Missing data | 5.91*** | 3.08 | 1.83 | 1.86 |
|  | (2.02 - 17.32) | (0.70 - 13.53) | (0.42 - 8.00) | (0.45 - 7.65) |
| HIV knowledge (base = <4 correct out of 5) |  |  |  |  |
| 4 out of 5 correct | 0.67* | 0.58 | 1.03 | 1.14 |
|  | (0.42 - 1.07) | (0.27 - 1.24) | (0.54 - 1.95) | (0.64 - 2.04) |
| All correct | 0.84 | 1.47 | 0.88 | 1.31 |
|  | (0.51 - 1.38) | (0.69 - 3.16) | (0.45 - 1.71) | (0.72 - 2.40) |
| Missing data | 3.99** | 0.65 | 1.13 | 0.26 |
|  | (1.21 - 13.17) | (0.09 - 4.60) | (0.19 - 6.61) | (0.02 - 3.08) |
| Partner type (base = married/cohabiting) |  |  |  |  |
| Main partner | 0.34*** | 2.06 | 0.56* | 1.51 |
|  | (0.20 - 0.59) | (0.70 - 6.13) | (0.29 - 1.08) | (0.71 - 3.24) |
| Casual partner | 0.23*** | 3.62** | 1.01 | 9.77*** |
|  | (0.12 - 0.44) | (1.21 - 10.87) | (0.54 - 1.88) | (4.12 - 23.19) |
| Missing data | 0.33 | 6.69 | 3.20 | 28.15*** |
|  | (0.05 - 2.44) | (0.45 - 98.87) | (0.40 - 25.67) | (3.61 - 219.39) |
| Partnership length (base = <1 month) |  |  |  |  |
| 2-6 months | 1.57 | 0.82 | 0.44** | 0.97 |
|  | (0.81 - 3.03) | (0.42 - 1.63) | (0.19 - 0.99) | (0.50 - 1.85) |
| 6-12 months | 1.21 | 0.47** | 0.33*** | 0.63* |
|  | (0.60 - 2.42) | (0.22 - 0.97) | (0.17 - 0.67) | (0.37 - 1.06) |
| >1 year | 2.11*** | 0.42** | 0.53** | 0.49*** |
|  | (1.21 - 3.68) | (0.19 - 0.92) | (0.33 - 0.87) | (0.30 - 0.80) |
| Missing data | 2.66** | 2.11 | 0.28* | 0.39* |
|  | (1.10 - 6.47) | (0.80 - 5.52) | (0.07 - 1.10) | (0.14 - 1.08) |
| Know partner’s HIV status | 0.81 | 0.67 | 0.81 | 0.66* |
|  | (0.55 - 1.21) | (0.34 - 1.33) | (0.50 - 1.31) | (0.41 - 1.05) |
| Constant | 0.49 | 0.05** | 0.72 | 0.05*** |
|  | (0.09 - 2.80) | (0.00 - 0.85) | (0.10 - 5.23) | (0.01 - 0.47) |
|  |  |  |  |  |
| Observations | 980 | 961 | 966 | 982 |

**Notes**: Adjusted odds ratios presented

*** p<0.01, ** p<0.05, * p<0.1

95% Confidence Intervals in parentheses

All analyses are adjusted to account for the complex study design and non-response.
