# Supplementary material for: Sexual Behaviour of Men and Women within Age-Disparate Partnerships in South Africa: Implications for Young Women's HIV Risk
Source: PLoS One. 2016 Aug 15;11(8):e0159162. doi: 10.1371/journal.pone.0159162 (PMC4985138; doi:10.1371/journal.pone.0159162)
Supplement: S1 Text — (DOCX) [file pone.0159162.s012.docx]

**S1 Text.** Relevant Questions from the Third National Communication Survey, 2012 used in our analysis

Listed below is the exact wording of the questions from the Third National Communication Survey that we used in our analysis. The list does not reflect all the questions asked in the survey or the ordering of the questions.

**Assets**

| Please tell me which of the following are presently in your household that are in working order [THIS HOUSEHOLD HERE] | |
| --- | --- |
| - Microwave oven |  |
| - Flush toilet in house or on plot |  |
| - Washing machine – automatic/ semi-automatic/ twin tub |  |
| - Built-in kitchen sink |  |
| - Water in home or on stand |  |
| - Electricity in the household |  |
| - One or more motor vehicles in household |  |

**Partnership related questions**

- How old is she/he now?
- When was the first time that you had sex with him/her?
- How would you describe your relationship with him/her?
- In the past year did you give this person gifts or money in order to have sex with him/her? [No; Yes, money; Yes, gifts]
- In the past year did you receive gifts or money from this person in order to have sex with him/her? [No; Yes, money; Yes, gifts]
- When was the most recent/last time that you had sex with him/her?
- Do you expect to have sex with him/her again?
- Did you use a condom the last time you had sex with this person?
- When you have sex with this person, how often do you use a condom?
- How many alcoholic drinks had you consumed when you had sex with this person the last time?
- Do you know this person’s HIV status?

**HIV knowledge questions/statements**

- Is it possible to cure HIV/AIDS?
- To prevent getting HIV you have to use condoms every time you have sex with someone.
- HIV is passed on most easily during the first 6 weeks after a person gets infected.
- A man who is circumcised does not need to use condoms to prevent getting HIV
- A person with HIV can look healthy
